# Personality and learning styles in relation to attitudes towards interprofessional education: a cross-sectional study on undergraduate medical students during their clinical courses

**DOI:** 10.1186/s12909-020-02327-7

**Published:** 2020-10-31

**Authors:** Caroline Olsson, Hanna Lachmann, Susanne Kalén, Sari Ponzer, Cecilia Mellstrand Navarro

**Affiliations:** 1 Department of Clinical Science and Education, Södersjukhuset, Karolinska Institutet, Forskningscentrum, Sjukhusbacken 10, Södersjukhuset, SE-118 46 Stockholm, Sweden; 2 Department of Health Sciences, The Swedish Red Cross University College, Huddinge, Sweden; 3 Department of Learning, Informatics, Management and Ethics, Karolinska Institutet, Stockholm, Sweden; 4 Department of Clinical Science and Education, Södersjukhuset, Karolinska Institutet, Stockholm, Sweden

**Keywords:** Interprofessional education, Undergraduate medical education, Personality, Learning styles, Big five inventory

## Abstract

**Background:**

Interprofessional Education (IPE) is now included in curricula in universities worldwide. It is known that there are differences in attitudes towards IPE among students, but less is known regarding how students’ personalities and learnings styles correspond with those attitudes. The aim of this study was to investigate whether personality traits and learning styles have any impact on medical students’ attitudes towards IPE.

**Methods:**

Seventy nine medical students in their 9th term (63% females, mean age 29 years) were questioned regarding their attitudes towards IPE according to the Interdisciplinary Education Perception Scale questionnaire, the Kolb’s learning style and Big Five Inventory questionnaires. For all three instruments we used the Swedish translated versions.

**Results:**

When investigated with a logistic regression, adjusting for age and gender, there were no significant associations between Big Five inventory, Kolb’s learning style and IEPS, except for the Reflective-Pragmatic learning style that was moderately associated with a higher IEPS score.

**Conclusion:**

There was no clear correlation between personality, learning style and attitude towards IPE as measured by the IEPS among medical students in our study population. Further investigations would benefit from a combination of qualitative and quantitative design.

**Supplementary Information:**

The online version contains supplementary material available at 10.1186/s12909-020-02327-7.

## Introduction

Interprofessional collaboration between different health care professionals is a strategy meant to improve patients’ medical outcome and increase patient safety. The idea is that when different health care professionals work together in well-functioning teams, they all contribute with specific knowledge adding up to a holistic approach [1]. However, it may be difficult to achieve good interprofessional teamwork. In an attempt to meet this challenge, universities have developed Interprofessional Education (IPE) learning activities to offer students opportunities to train and learn collaboration skills [2, 3]. IPE promotes safe patient care (World Healt h[4]) and has been widely implemented [5]. IPE has been defined as “members or students of two or more professions associated with health or social care, engaged in learning with, from and about each other” ([2], p. 43). IPE is currently included in curricula at many universities worldwide (C. L. [6]); however, “there is no generalizable theory which can explain how, why or when learning through IPE is successful” (C. L. [6], p. 2). Allvin et al. stated in a study protocol from 2020 that IPE includes many aspects of collaboration and learning activities on different levels. This complexity of IPE-field provides a challenge in terms of research and they conclude that previous research consists of contradictory results and that “the effects of IPE on learning outcomes across all health professions are inconclusive” ([7], p. 2). Although the use of IPE as a teaching method in healthcare education has increased during the last 20 years, it is important to recognize that students’ attitudes toward IPE seem to vary [8]. There are gender differences in attitudes [9], as well as differences based on what educational program the students attend. Wilhelmsson et al. [9] concluded that female students were more positive towards IPE/teamwork than male students, regardless of the educational program they were attending. Another finding was that nursing students had a more positive attitude to teamwork and collaboration compared to medical students. Students that participate in courses in IPE ward seem to be more positive to IPE and to collaboration with other professions (C. L. F [10].).

In 2017, Visser et al. carried out a systematic review to identify how IPE was perceived by medical students, residents, and nursing students in a clinical setting. Their conclusion was that there are three levels of explanations for successful IPE activities: the organizational, processual, and individual levels. In their study, Visser [6] identified a need for more research on the individual level. Our article is an attempt fill this knowledge gap by analyzing different factors on a personal level in order to increase the understanding of students´ attitudes towards IPE. Learning styles have been discussed as one of the components that can have impact on students’ performance [11, 12]. Personality is another aspect that is likely to affect learning processes as well as attitudes towards IPE. A recent study [13] from Israel found positive correlations between nursing students´ perception of actual cooperation with other professions (IC) and the personality traits; agreeableness, conscientiousness, and openness to new experiences. Regarding medical students there is a lack of previous studies examining the relationship between personality traits and attitudes towards IPE. In two different systematic reviews examining IPE personality traits were not included [14, 15]. We would argue that the influence of learning styles as well as personality traits regarding attitudes towards IPE and interprofessional collaboration is a research field with knowledge gaps. To the best of the authors’ knowledge, there are no published studies exploring the correlation between learning styles, personality traits and attitudes towards IPE.

The aim of this study was to investigate whether personality traits and learning styles have any impact on medical students’ attitudes towards IPE and interprofessional collaboration.

## Methods and participants

The curriculum of the medical program at Karolinska Institutet is not designed as interprofessional but some interprofessional activities are included throughout the program. The longest IPE period is a mandatory 2 weeks interprofessional clinical rotation, that takes place during their 8th term at a ward or in an emergency room setting [16]. This rotation is an IPE course with specific learning outcomes related directly to IPE. During these interprofessional clinical rotations students experienced active collaboration with nursing students, and sometimes also with physiotherapist- and occupational therapist students, in real patient care and the students discuss and solve upcoming clinical problems together [17, 18]. At the medical program and the other health care related educational programs at Karolinska Institutet IPE is in line with the descriptions made by CAIPE, and they state that IPE is “occasions when two or more professions learn with, from and about each other to improve collaboration and the quality of care” [19].

The null hypothesis of this cross-sectional cohort study was a priori defined as that there is no correlation between personality traits, learning styles and medical students’ attitudes towards IPE and interprofessional collaboration. In the fall of 2012 all 6th-semester medical students at Karolinska Institutet were invited to participate in a study that used the Contextual Activity Sampling System (CASS) for data collection using mobile devices [20–22]. All students were informed both orally and written, during their first week in 6th semester out of eleven about the research project’s aim and design plus what participation would imply. Further they were informed about that participation were voluntary and that they could decline at any time without consequences, neither for their grades nor course participation. Informed consent was obtained by all participants.

When constructing experience-sampling questionnaires it is important to be cautious [23] to avoid reactivity among the respondents concerning frequent sampling. Therefore, the total number and frequency of questionnaires to be used during this study were thoroughly discussed in the research group based on previous research on the topic as described by Van Berkel et al. and Heron et al. [24, 25]. After consensus in the study group, questionnaires were decided to be sent via mobile phones with follow-ups every 3rd week during the 6th semester, and this data has been published elsewhere [26]. Background data (gender and age) were also collected through the CASS on the first occasion.

During the 9th semester, students´ attitudes towards IPE were investigated by using the Interdisciplinary Education Perception Scale (IEPS) (B [27].). The Kolb’s learning style questionnaire [28] and the Big Five Inventory (BFI) questionnaire were used regarding learning styles and personality traits respectively [29]. The IEPS is a tool originally designed to measure attitudes towards interprofessional education [30]. In this study we used the Swedish translated version that has been adapted to consist of three subscales measured with a Likert scale ranging from 1 = strongly disagree to 6 = strongly agree (B [27].). The *competency and autonomy* subscale refers to respect and trust among professions, positive attitudes about goals and contributions, and attitudes about students’ training and competence. The *perceived need for cooperation* subscale refers to students’ attitudes about the need to cooperate and to learn from other professions. The *perception of actual cooperation* subscale refers to students’ attitudes about teamwork, positive interprofessional relationships, and willingness to collaborate with those in other professions. The IEPS total score is a sum of the three subscores. The competency and autonomy subscale is the sum of responses to five questions with a maximum score of 30. The perceived need for cooperation is the sum of responses to two questions with a maximum score of 12, and the perception of actual cooperation subscale is the sum of responses to five questions with maximum score of 30. The maximum total sum score of 72 indicates a more positive response to interprofessional cooperation. For this study, the IEPS results were judged as positive (61–72 points) or negative (51–60 points) towards interprofessional collaboration.

According to Kolb’s experiential learning theory, learning is viewed as a four-stage cycle [31]. First, immediate and concrete experiences serve as a basis for observation. Next, the individual reflects on these observations and begins to build a general theory of what this information might mean. In the next step, the learner forms abstract concepts and generalizations based on the hypothesis. Finally, the learner tests the implications of these concepts in new situations. Hereafter, the learning process continues in the four stages as an endless spiral of the experiential process. The learning styles described by Kolb are based on two major dimensions: active/reflective and abstract/concrete. In the Swedish version that we used in this study, these are presented as two partially redefined variables: Emotive-Rational and Reflective-Pragmatic. These two variables are independent of each other and have good reliability, expressed both as homogeneity and stability [28]. Kolb’s learning style inventory dimensions of Emotive-Rational and Reflective-Pragmatic have been calculated in this study according to the model defined by Marke and Cesarec [28]. For more information, see Appendix 1 (Additional file 1).

The Big Five Inventory (BFI) is a well-established and validated instrument used to measure personality traits [29]. The major advantages of the BFI is that it is easily understood by respondents because it is derived from ordinary people’s vocabulary, it seems to capture humans’ personalities in different cultures, and it can be used to predict important life events. Five types of personality traits are used in the model. Extraversion refers to someone who is social, active, and likes to have fun. Agreeableness represents someone who is helpful, forgiving, and honest. Conscientiousness refers to someone who thinks before acting and is good at organizing and prioritizing. Neuroticism refers to someone who feels anxious, nervous, sad, and tense. Openness to experience describes a person who is curious, interested in new things, and intellectual [29]. The BFI has been translated, validated and used in a Swedish context [32]. The BFI consists of 44 items on a 5-point Likert scale, where 1 indicates “I strongly disagree”; 2, “I disagree”; 3, “I neither agree nor disagree”; 4, “I agree”; and 5, “I agree strongly.” Each personality dimension’s overall score is calculated by summing up the Likert scale scores of the 8–10 assertions specific for each dimension [32].. The personality dimension scores were dichotomized into the following categories with the median value of the whole population defining the cut-off; extraversion 13–28 = no, 29–40 = yes, agreeableness 27–35 = no, 36–45 = yes, neuroticism 9–20 = no, 21–35 = yes, conscientiousness 21–35 = no, 36–45 = yes, openness 21–35 = no, 36–50 = yes.

### Statistics

Statistical analyses were performed using software packages of SPSS version 23. The cohort size was defined by convenience sampling, including one class of medical students. To compare proportions of categorical variables, the Chi Squared test was used. Bonferroni’s correction was used for multiple comparisons. To compare numerical values, a Student’s t-test was used. All comparisons were considered significant if *p* < 0.05 in two-sided tests.

The main outcome of IEPS is a numerical ordinal value and was presented as means and corresponding standard deviation. The correlations between IEPS and age, gender, previous experience with IPE, Kolb’s learning styles, and personality according to BFI, respectively, were tested in a logistic regression model in all individuals with complete data. Other confounders were not considered in the analysis. Results are presented as crude measures, univariate analysis and multivariable analysis adjusted for age, gender, KOLB, and BFI. The results are presented as odds ratios for crude and adjusted measures for being positive to IPE. Statistical significance is presented as 95% confidence intervals and *p*-values.

#### Ethics

This study was performed according to the Helsinki Declaration (World Medical Association Declaration of Helsinki. Ethical Principles for Medical Research Involving Human Subjects, 1964). The Regional Ethical Review Board in Stockholm reviewed the study and stated that no ethical permission was required per Swedish law (Dnr: 2010/1606–31/5, 2012/1227–32).

## Results

Ninety-eight students out of 136 eligible individuals agreed to participate in the study, out of whom 79 responded to the questionnaires and were included in the current analysis. The mean age of the participants was 29 years (SD = 5.3, range 23–46 years) and 62% (*n* = 50) were females.

Table 1 shows the mean scores for the IEPS. Table 2 presents the IEPS subscales. Generally, the scores were relatively high, and the subscale perceived need for cooperation reached almost a maximum score (11.7 of 12 possible). There were no differences between genders.

**Table 1:** Mean scores of participants on the Interdisciplinary Education Perception Scale in a study investigating personality and learning styles in relation to attitudes towards interprofessional activities among 79 undergraduate medical students

	Item	Males Mean (SD) ***range***	Females Mean (SD) ***range***	Total Mean (SD) ***range***
1	Individuals in my profession are well-trained *(1 missing)*	5.4 (0.49) *5–6*	5.4 (0.65) *4–6*	5.4 (0.59) *4–6*
2	Individuals in my profession are able to work closely with individuals in other professions	5.1 (0.59) *4–6*	4.8 (0.71) *3–6*	4.9 (0.69) *3–6*
3	Individuals in my profession are very positive about their goals and objectives	4.9 (0.64) *4–6*	4.8 (0.62) *4–6*	4.8 (0.63) *4–6*
4	Individuals in my profession need to cooperate with those in other professions	5.9 (0.31) *5–6*	5.8 (0.55) *4–6*	5.8 (0.51) *4–6*
5	Individuals in my profession are very positive about their contributions and accomplishments	4.9 (0.59) *4–6*	4.9 (0.78) *3–6*	4.9 (0.71) *3–6*
6	Individuals in my profession must depend on the work of people in other professions	5.8 (0.47) *4–6*	5.9 (0.42) *4–6*	5.8 (0.43) *4–6*
7	Individuals in my profession trust each other’s professional judgment	4.7 (1.0) *2–6*	4.8 (0.73) *3–6*	4.8 (0.85) *2–6*
8	Individuals in my profession are extremely competent *(1 missing)*	4.7 (0.71) *3–6*	4.5 (0.75) *3–6*	4.6 (0.73) *3–6*
9	Individuals in my profession are willing to share information and resources with other professionals	4.9 (0.70) *3–6*	4.8 (0.74) *3–6*	4.9 (0.72) *3–6*
10	Individuals in my profession have good relations with people in other professions *(1 missing)*	4.4 (0.73) *3–6*	4.5 (0.69) *3–6*	4.4 (0.70) *3–6*
11	Individuals in my profession think highly of other related professions	3.9 (0.92) *2–6*	4.2 (0.65) *3–6*	4.1 (0.76) *2–6*
12	Individuals in my profession work well with each other	4.5 (0.82) *3–6*	4.8 (0.65) *4–6*	4.7 (0.73) *3–6*

**All comparisons between genders were insignificant (*p*** **> 0.05), Student’s t-test**

**Table 2:** Students Interdisciplinary Education Perception Scale (IEPS) subscales investigated in a study investigating personality and learning styles in relation to attitudes towards interprofessional activities among 79 undergraduate medical students. Maximum possible scores: total 72; 1st subscale 30; 2nd subscale 12; 3rd subscale 30

	N	Males Mean (SD) ***range***	Females Mean (SD) ***range***	Total Mean (SD) ***range***
**IEPS total**	76	59.2 (4.0) *52–68*	59.1 (4.4) *51–72*	59.1 (4.2) *51–72*
**IEPS subscales**				
1. Competency and autonomy	77	24.6 (2.0) *21–29*	24.3 (2.3) *20–30*	25.5 (2.2) *20–30*
2. Perceived need for cooperation	79	11.7 (0.70) *9–12*	11.6 (0.85) *8–12*	11.7 (0.8) *8–12*
3. Perception of actual cooperation	78	22.9 (2.9) *17–30*	23.1 (2.4) *19–30*	23.0 (2.6) *17–30*

**All comparisons between genders were insignificant (*p*** **> 0.05), Student’s t-test**

Table 3 summarizes the participants’ personality dimensions according to the BFI questionnaire. There were significant differences between genders in three out of five dimensions, with female students scoring significantly higher in the agreeableness, neurotic and conscientiousness dimensions.

**Table 3:** Personality dimensions according to the Big Five questionnaire in a study investigating personality and learning styles in relation to attitudes towards interprofessional activities among 79 undergraduate medical students. The results presented refer to number of respondents with high scores on the corresponding dimensions of the BFI (e.g yes for Agreeableness/Neuroticism etc.) and Total % the percentage of high scores on the BFI in relation to the entire study population (*n* = 79)

	Males	Females	Total	Total %
Extraversion	10	19	29	37
Agreeableness (1 missing)	12*	33*	45	58
Neuroticism (2 missing)	8*	29*	37	48
Conscientiousness (4 missing)	6*	30*	36	48
Openness (1 missing)	19	22	41	53

*Significant difference between genders (*p* **< 0.05), Chi Square test with the Bonferroni correction**

Table 4 shows the Kolb’s learning inventory. Female students had higher scores for the dimension Reflective-Pragmatic compared to male students (*p* = 0.024).

**Table 4:** Kolb’s learning inventory in two dimensions: Emotive-Rational and Reflective-Pragmatic, in a study investigating personality and learning styles in relation to attitudes towards interprofessional activities among 79 undergraduate medical students

	Males Mean (SD) ***range***	Females Mean (SD) ***range***	Total Mean (SD) ***range***
Emotive-Rational	42.9 (8.9) *29–60*	43.6 (8.7) *28–66*	43.3 (8.7) *28–66*
Reflective-Pragmatic	39.5 (9.9) 29–65*	45.3 (9.6) 31–70*	43.0 (10.1) *29–70*

*Significant difference between genders (*p* **< 0.05), Student’s t-test**

There were complete data regarding IEPS for 76 students, out of which 49 students (64%) scored 51–60 points on the IEPS, which was interpreted as a negative attitude towards IPE, whereas 27 students (36%) scored 61–72 points, which was considered a positive attitude towards IPE. When investigated using a logistic regression adjusting for age and gender, a moderate association was found between the Reflective-Pragmatic learning style, and a higher IEPS score, as shown in Table 5. No other relationship was found between the main outcome IEPS and Kolb’s learning style or BFI in either the univariate or the adjusted analysis.

**Table 5:** A logistic regression model investigating the relationship between IEPS and KOLB learning style, BFI, gender, and age, in a study investigating personality and learning styles in relation to attitudes towards interprofessional education (IPE) among undergraduate medical students. Results are expressed as an odds ratio (OR) for being positive to IPE in a univariate and multivariable analysis

Variable			Crude measures		Univariable			Multivariable		
								Adjusted for all variables*		
					OR	95% CI	***p***-value	OR	95% CI	***p***-value
			**n**	**%**						
**KOLB**	**EMORAT**	**1**	16	27	Reference			Reference		
		**2**	16	27	1.000	0.239–4.184	1.00	1.473	0.197–11.032	0.706
		**3**	28	47	1.500	0.408–5.518	0.542	3.086	0.469–20.291	0.241
	**REPRA**	**1**	18	30	Reference			Reference		
		**2**	18	30	3.500	0.825–14.848	0.089	8.049	1.206–53.707	0.031**
		**3**	24	40	2.429	0.678–8.701	0.173	4.094	0.612–27.374	0.146
**BFI**	**Extraversion**	**Yes**	24	40	Reference			Reference		
		**No**	36	60	1.857	0.624–5.530	0.266	1.758	0.416–7.425	0.443
	**Agreeableness**	**Yes**	37	62	Reference			Reference		
		**No**	23	38	1.725	0.550–5.412	0.350	3.115	0.681–14.254	0.143
	**Neuroticism**	**Yes**	28	47	Reference			Reference		
		**No**	32	53	0.351	0.112–1.098	0.072	0.315	0.075–1.319	0.114
	**Conscientiousness**	**Yes**	30	50	Reference			Reference		
		**No**	30	50	1.833	0.616–5.453	0.276	1.251	0.276–5.666	0.772
	**Openness**	**Yes**	33	55	Reference			Reference		
		**No**	27	45	0.545	0.184–1.613	0.273	0.347	0.078–1.549	0.165
**Gender**	**Male**		23	38	Reference			Reference		
	**Female**		37	62	1.11	0.370–3.338	0.851	0.607	0.101–3.665	0.587
**Age**	**< 27**		26	43	Reference			Reference		
	**27–30**		20	33	0.684	0.125–1.929	0.308	0.301	0.062–1.476	0.139
	**> 30**		14	23	0.491	0.193–2.419	0.556	0.259	0.034–1.947	0.189

*CI* Confidence Interval

*BFI* Big Five Inventory

*IPE* Interprofessional Education

*IEPS* Interdisciplinary Education Perception Scale

*Logistic regression adjusted for age, gender, KOLB, and BFI

** Statistically significant

## Discussion

The aim of this study was to investigate associations between learning styles, personality and medical students’ attitudes towards IPE and interprofessional collaboration. Visser et al. [6] mentioned the lack of research investigating individual factors in relation to IPE. Learning styles and personality traits are examples of factors on an individual level, which are likely to have an impact on students’ attitudes towards IPE and interprofessional collaboration. In line with this assumption, it was hypothesized that both learning styles and personality traits would have an impact on attitudes toward IPE; however, there were no relevant associations found in the regression model investigating KOLB’s and BFI’s association with IEPS. Although there were no significant associations in the data, some findings are still interesting in relation to previous research.

The data were collected in 2014 alongside a longitudinal study with medical students at Karolinska Institutet [33, 34]. The demographic data showed that the gender distribution was in line with the current male-female ratio of students in Sweden and at Karolinska Institute where the study was conducted. The sample is representative, and the findings are generalizable from a gender perspective. The participants were all familiar with the CASS method of data collection, and the response rate was relatively high, which further supports the appropriateness and generalizability of the findings.

In several previous studies, gender did seem to play a role in attitudes towards IPE [35, 36], showing that female students were more positive towards IPE compared to male students. In contrast, in our study, no difference was found between genders for the IEPS results, a finding supported by a study by Williams et al. (Brett [37]).

In our study, IEPS scores were found to be relatively high, and the subscale “perceived need for cooperation” reached almost a maximum score. The purpose of our study was probably obvious to the study participants, and that may have introduced bias in our findings. Students may have responded to our questions in a manner that they thought would please us, thus producing a higher IEPS score than students normally would achieve. However, the fact that all CASS answers were anonymized minimized the risk of such bias. Moreover, in a population with a generally positive attitude towards IPE, there may exist little differences between groups that are not discernable due to ceiling effects in our measurement tool. A larger study, and/or another study design would be needed to clarify these circumstances. Psychometric robustness of instruments to evaluate IPE has been reported to be questionable [38]. Problems relating to the IEPS involve that use of the IEPS by various authors demonstrates considerable variation in the application of the original test scoring protocol. In some papers, raw scores have been used for analysis rather than factor or subscale scores and total scores [38]. We have used a dichotomized result. The cut-off was arbitrarily set at 60, which may be subject for discussion and/or criticism. Our study aim was to investigate an association between IPE attitudes and learning styles with a quantitative approach. After discussion in the study group, we decided for the current methodology, which has its weaknesses but still represents an inventory of strong associations between different psychometric measures in the population under study.

In a study from Marke and Cesarec [28], females were found to be more emotive in their learning modes independent of profession and not correlated to age. They also showed that students of social work and psychology were the most emotive and most prone to reflection, while medical students had more pragmatic and rational learning modes. Heffler and Sandell, who studied learning styles among psychotherapy students, found that men were more rational than women. However, this finding was only significant in one of three cohorts studied [39]. The findings from our study showed that female medical students had a significantly more reflective pragmatic learning style than male students; however, no difference in the Emotive-Rational learning style between genders was observed. The findings regarding relations between gender and learning styles are contradictory in the literature and we can only conclude a need for further investigation.

Well-established and validated instruments were used to measure students’ learning styles, personality traits, and attitudes towards IPE by using the Swedish version of IEPS (B [27].). Even so, there are some limitations to this study that should be recognized. First, the number of participants was relatively low and all participants were medical students from the same semester and from one single university. Future research would benefit from using a larger sample, with students from different educational programs and / or time periods that would allow a comparative approach. Significant associations may have been found using a larger or different sample. Second, although well-established and validated instruments were used to measure learning styles, personality traits, and attitudes towards interprofessional education activities, it should be noted that these are all complex processes that can be difficult to investigate using a quantitative design. Future research would benefit from a combination of quantitative and qualitative research methods.

## Supplementary Information

**Additional file 1.** Appendix 1: English Summary of Marke and Cesarecs Swedish version of Kolb’s Learning Style Inventory

## Data Availability

The dataset supporting the findings and the conclusions of this article are available.

## Abbreviations

BFI: Big five inventory
CAIPE: The centre for advancement of interprofessional education
CASS: Contextual activity sampling system
IPE: Interprofessional education
IPES: Interdisciplinary education perception scale
