# Supplementary material for: Personality and learning styles in relation to attitudes towards interprofessional education: a cross-sectional study on undergraduate medical students during their clinical courses
Source: BMC Med Educ. 2020 Oct 31;20:398. doi: 10.1186/s12909-020-02327-7 (PMC7603747; doi:10.1186/s12909-020-02327-7)
Supplement: Supplementary file 1 — Additional file 1. Appendix 1: English Summary of Marke and Cesarecs Swedish version of Kolb’s Learning Style Inventory [file 12909_2020_2327_MOESM1_ESM.docx]

Appendix 1: English Summary of Marke and Cesarecs Swedish version of Kolb’s Learning Style Inventory

Swedish title: Erfarenhetsinlärning och lärstilar. Analys och standardisering av Kolb’s Learning Style Inventory i svensk version.

The report contains a longer summery in English by Sven Marke and Zvonimir Cesarec originally published in 2007 (Marke & Cesarec, 2007).

Marke and Cesarecs report contains a psychometric analysis given of the Swedish-language version of the Learning Style Inventory (LSI-2) that we used in our study. In addition to scale analysis, the work comprises validity testing through comparison between different groups, the learning style’s correlation with personality traits and an application of work on group dynamics.

Their data consists of students (e.g physicians (n=1010) and social work students (n=1237) and some minor groups of other students) and three types of validity testing were conducted (e.g group comparisons, relationship to other instruments, group dynamic application. The results were interpreted as in line with previous research and that the validity testing could confirm the validity of the Swedish version of the test.

Marke, S., & Cesarec, Z. (2007). *Erfarenhetsinlärning och lärstilar : analys och standardisering av Kolb's Learning Style Inventory i svensk version : with an english summary / Experiential learning and learning styles: analysis and standardization of Kolb's Learning Style Inventory in Swedish version*. Stockholm: The Swedish National Board of Institutional Care, Statens institutionsstyrelse.
